# Sensitive Detection of Oral Leukoplakia: Analyzing P90 Biomarkers in Saliva and Tissue

**DOI:** 10.3390/bios14060281

**Published:** 2024-05-30

**Authors:** Hsiao-Hsuan Wan, Haochen Zhu, Chao-Ching Chiang, Jian-Sian Li, Fan Ren, Cheng-Tse Tsai, Yu-Te Liao, Dan Neal, Joseph Katz, Josephine F. Esquivel-Upshaw

**Affiliations:** 1 Department of Chemical Engineering, University of Florida, Gainesville, FL 32611, USA; hwan@ufl.edu (H.-H.W.); zhu.haochen@ufl.edu (H.Z.); cchiang@ufl.edu (C.-C.C.); jiansianli@ufl.edu (J.-S.L.); fren@che.ufl.edu (F.R.); 2 Department of Electronics and Electrical Engineering, National Yang Ming Chiao Tung University, Hsinchu 30010, Taiwan; datsai1125.ee09@nycu.edu.tw (C.-T.T.); yudoliao@nycu.edu.tw (Y.-T.L.); 3 Department Surgery, University of Florida, Gainesville, FL 32611, USA; dneal@ufl.edu; 4 Department of Oral and Maxillofacial Diagnostic Sciences, University of Florida, Gainesville, FL 32611, USA; jkatz@dental.ufl.edu; 5 Department of Restorative Dental Science, Division of Prosthodontics, University of Florida, Gainesville, FL 32611, USA

**Keywords:** oral cancer detection, oral leukoplakia, P90, high sensitivity, salivary biomarkers, MOSFET, tissue

## Abstract

Oral cancer represents a significant global public health challenge, contributing substantially to the incidence and mortality of cancer. Despite established risk factors such as tobacco use and alcohol consumption, early detection remains crucial for effective treatment. This study introduces a novel approach using a transistor-based biosensor system for detecting the P90 (CIP2A) protein. We tested the presence of CIP2A in human leukoplakia samples, which can undergo malignant conversion into aggressive oral squamous cell carcinoma. The method used commercially available glucose test strips functionalized with P90 antibodies, providing high sensitivity and a low limit of detection which was five orders lower than that of commercial ELISA kits. A specially designed printed circuit board (PCB) facilitated accurate measurements, and the device’s performance was optimized through characteristic tests. Human sample testing validated the biosensor’s effectiveness in distinguishing samples after cell lysis. This study contributes to advancing accurate and cost-effective diagnostic approaches for oral pre-cancer and cancer tissues.

## 1. Introduction

Oral leukoplakia (OL), as defined oral by the World Health Organization, is a condition where a white patch of oral mucosa cannot be erased or classified otherwise, either clinically or histopathologically. OL is among the most common oral potentially malignant disorders (OPTMs), defined as any oral mucosal abnormality that is associated with a statistically increased risk of developing oral cancer [1]. Oral cancer stands as a considerable global public health concern, constituting a substantial proportion of the overall incidence of cancer. Annually, millions of individuals receive diagnoses of oral cancer, contributing significantly to the global cancer burden. The American Cancer Society reported an anticipated 54,540 new cases of oral cavity or oropharyngeal cancer in 2023, with an estimated 11,580 fatalities attributed to these specific cancer types in the same year [2]. The utilization of tobacco and the consumption of alcohol are recognized as established risk factors for oral cancer, with approximately 75% of all reported cancer cases being linked to these specific exposures [3,4,5,6]. Additional factors, including human papillomavirus (HPV) infection, dietary habits, nutritional considerations, gender, and exposure to radiation, contribute to an elevated likelihood of being diagnosed with oral cancer [7,8,9]. Cancers also result in losses in productivity, with oral cancer causing an economic impact of approximately $0.74 billion in India and $112,308 in South Africa [10].

Early detection of oral cancer is crucial for successful treatment [11,12]. The primary diagnostic methods for oral cancer involve clinical examinations, biopsies, imaging studies (X-rays, CT scans, and MRI scans), and endoscopy [13,14,15]. Clinical examination entails visual inspection by a healthcare professional to identify visible abnormalities [16]. A biopsy is essential for confirming the presence of cancerous cells and determining the type and stage of the cancer [17]. Imaging studies, including X-rays and CT scans, provide detailed images aiding in of identification the tumor [18]. These common diagnostic approaches facilitate early detection, which is crucial for effective treatment and improved outcomes in oral cancer cases. While there are numerous diagnostic methods, they have inherent limitations. Clinical examinations may be subjective and limited to surface observations, potentially missing deeper lesions. Biopsies, while definitive, are invasive and carry a risk of sampling errors due to the small tissue sample obtained [11,19,20]. Imaging studies can produce false positives/negatives and expose patients to radiation [21]. Acknowledging these limitations emphasizes the ongoing need for research to refine existing methods and explore complementary approaches for more accurate and comprehensive oral cancer diagnostics.

P90, alternatively identified as KIAA1524 and Cancerous Inhibitor of PP2A (CIP2A), exhibits elevated expression levels in both oral squamous cell carcinoma (OSCC) cell lines and tissues [22,23,24,25]. This protein is of considerable interest as a promising therapeutic target or a potential diagnostic marker, given the relatively low expression levels of CIP2A in normal tissues [26]. There are additional reports that imply the positive role of protein phosphate 2A (PP2A) in inflammatory lung diseases such as asthma and chronic obstructive pulmonary disease (COPD), and in heart function [27]. These effects are attributed to PP2A‘s inhibitory effects on the mediators of inflammation. For the analysis of CIP2A in saliva, enzyme-linked immunoassay (ELISA) test kits are readily accessible, and the development of immunosensors utilizing carbon nanotubes has commenced [28]. However, growing the nanotubes is time-consuming and also quite expensive. A recently introduced method for detecting CIP2A showed high sensitivity through the use of a transistor-based biosensor system. The P90 protein is treated with a PBS solution in this process, achieving a low limit of detection at 10^−15^ g/mL. Notably, the sensitivity of this method surpassed that of commercially available ELISA test kits [24]. While it exhibited high sensitivity and a low limit of detection, it is noteworthy that the standard calibration solution used in this method was PBS, rather than artificial saliva. Furthermore, human sample testing was not conducted. In contrast, our study established a calibration curve using P90 protein diluted in artificial saliva. A specially designed printed circuit board (PCB) was implemented for the detection of the concentrations of P90 protein. The accuracy of the newly developed method was demonstrated through testing human samples obtained from oral cancer patients.

## 2. Materials and Methods

**Figure 1:**
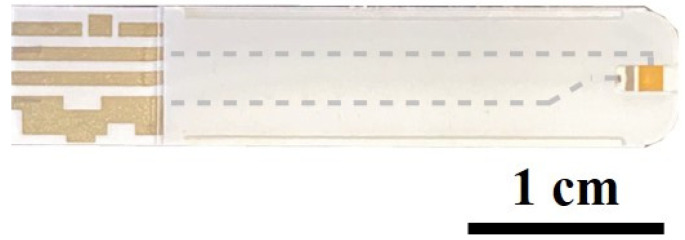
Schematic of the test strips.

**Figure 2:**
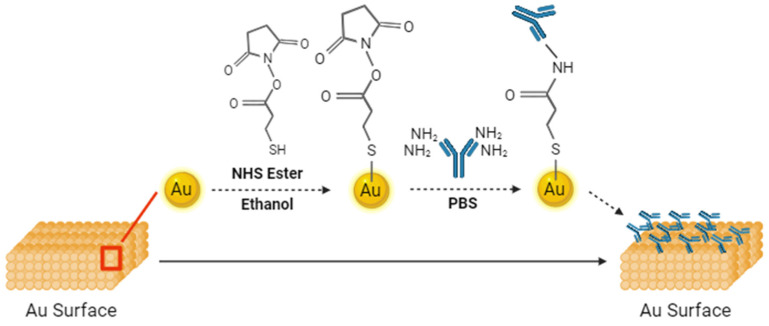
Functionalization steps used for the strips.

The test strips (Luvnshare Biomedical Inc., Hsinchu, Taiwan) used in this study are shown in Figure 1. They are similar to commercially available glucose test strips but without the enzyme. They have only bare electrodes, which we functionalized. The tip of the strips features microfluidic channels for injection of the sample. A gold-plated electrode is present on the tip, which underwent a sequence of functionalization processes with the P90 antibody. The functionalization step is shown in Figure 2. This functionalization enabled the strips to discern variations between samples. The initial step in the functionalization process was ozone treatment of the strips for a duration of 15 min, which effectively removed any carbon residues. Subsequently, a diluted ammonium hydroxide (NH_4_OH) solution was used to eliminate gold oxide. Following this surface cleaning step, deionized (DI) water was used to rinse the channels, and nitrogen was utilized for the drying process. The next phase involved preparing a 3-mercaptopropanyl-N-hydroxysuccinimide ester (NHS ester) solution, which was dissolved in ethanol. NHS ester has a three-carbon chain ending in a thiol group, with an attached N-hydroxysuccinimide ester. This compound was utilized for bioconjugation, offering a reactive site for selective coupling with amine-containing molecules. The strips were submerged in this solution and left to react for 2 h. The channels were then cleaned using DI water and nitrogen. Monoclonal CIP2A antibody 2G10-3B5 (Santa Cruz Biotechnology, Dallas, TX, USA) at a concentration of 20 μg/mL was injected into the channel, and the strips were sealed and stored in a disk at 4 °C for 18 h. Lastly, ethanolamine was used to deactivate the unfunctionalized groups, mitigating the risk of potential interference. The previous research provided confirmation of the functionalization of the antibody through uniform methodologies, as indicated by current–voltage and capacitance measurements [29,30,31]. CIP2A protein (MyBioSource, San Diego, CA, USA) was diluted into a series of concentrations with artificial saliva (Pickering Laboratories Inc., Mountain View, CA, USA) to establish the calibration curve.

**Figure 3:**
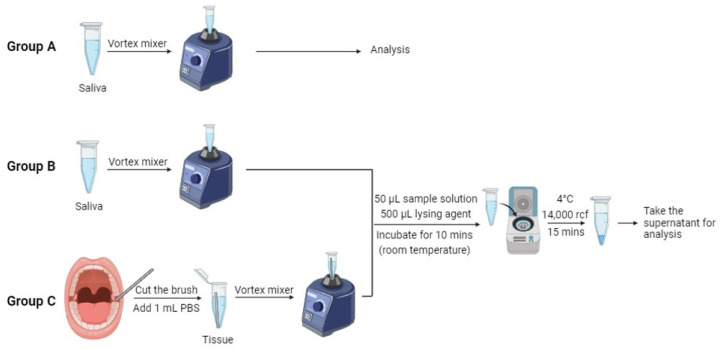
Procedure of collecting tissue samples and cell lysis. Group A consists of saliva samples that have not undergone cell lysis. Group B comprises saliva samples that have been lysed, while Group C comprises lysed tissue samples.

Seventeen human saliva samples and tissue samples were obtained from individuals, including both oral pre-cancer patients and healthy volunteers, through collaboration with the University of Florida’s Oral Pathology Clinic and Dental Clinical Research Unit. The age range of the healthy individuals was from 20 to 80 years old, while patients with inflammatory oral lesions (clinically diagnosed as leukoplakia) ranged from 40 to 80 years old. Table 1 displays comprehensive information regarding the patients examined in this study. We aimed to investigate whether age influenced the detection results. Brush kits (Andwin Scientific, Simi Valley, CA, USA) were used in the collection of tissue samples. These specimens were carefully preserved in a deep-freeze storage unit at −78 °C. In this study, we tested three groups of samples. Group A consisted of saliva samples without cell lysis, Group B comprised lysed saliva samples, and Group C comprised lysed tissue samples. The native lysis buffer used for the saliva and tissue samples was purchased from Thermo Fisher Scientific (Waltham, MA, USA). The procedures of sample collection and cell lysis are shown in Figure 3. Epithelial cells were obtained by turning the brushes against the mucosa inside the oral cavity. The head of the brushes was then cut and placed in a 1.5 mL microcentrifuge tube. After that, 1 mL of a 1× PBS buffer solution was added into the tube, and the tissue samples were suspended in the solution by a vortex mixer. In the procedure of cell lysis, the sample solution was initially combined with the native lysis buffer at a 1:10 ratio in a 1.5 mL microcentrifuge tube. Here, we took a 50 μL (1 drop) sample of the solution with 50 μL of the lysing agent, followed by thorough mixing using a vortex mixer. Subsequently, the mixture was incubated at room temperature for 10 min. Finally, the mixture was centrifuged at 14,000 rcf for 15 min at 4 °C to separate the resulting supernatant and pellet, which were then stored in a refrigerator for subsequent analysis.

**Figure 4:**
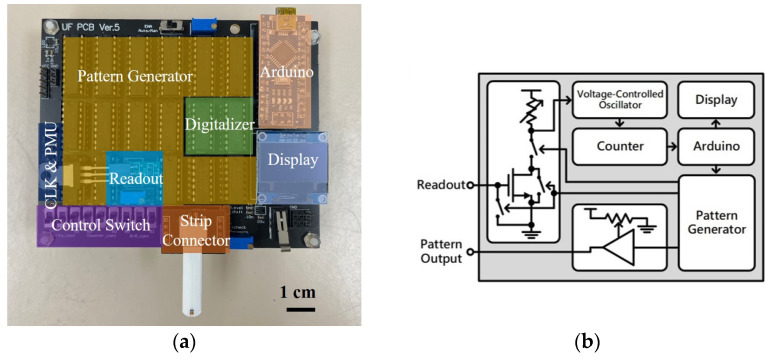
(**a**) Printed circuit board used in this work (**b**) Block diagram for the design of the printed circuit board.

The printed circuit board (PCB) used in this study is shown in Figure 4a. This portable detection device consists of the readout block (Readout), a pattern generator (Pattern Gen.), a digitalizer, a strip connector, an Arduino, a display, a control switch, and a system clock and power management unit (CLK and PMU). Figure 4b demonstrates the simplified circuit diagram of the PCB. The following paragraphs will explain how this device operates and provide details about the device.

The process for the sensor’s readout is as follows. First, the strip is connected to the strip connector while the Arduino is activated. The Arduino generates signals, triggering the pattern generator to generate a test pattern for measurement. The test pattern, passing through the strip, produces output signals of different magnitudes. This signal is then inputted into the gate terminal of the metal oxide semiconductor field effect transistor (MOSFET) in the readout block, where amplification occurs using the MOSFET and its drain-side potentiometer. The amplified readout signal is converted into a frequency signal by the voltage-controlled oscillator (VCO) in the digitalizer. This frequency signal is then counted at fixed intervals by the counter. When the VCO reads a higher voltage, resulting in a higher output frequency, the counter outputs a larger value. Conversely, when the VCO reads a lower voltage, resulting in a lower output frequency, the counter outputs a smaller value. Because the counter’s output varies with the MOSFET’s output voltage, this value can be used as a digital representation of the readout voltage. Finally, the counter’s output is processed by the Arduino and displayed on the mini-LCD screen, allowing users to directly read the numerical value to determine the concentration of the solution on the strip.

In each measurement, the device outputs multiple test patterns for repeated measurements, and the results are averaged to reduce measurement errors. To avoid the charge accumulation effect on the strip during the measurement process, the gate terminal of the MOSFET is grounded to release the accumulated charge on the strip and gate terminals after the measurement of each test pattern has been completed. This ensures the accuracy of each measurement. Additionally, this device has high adjustability. For example, the control switch can adjust the length of the test pattern and control the timing of the digitalizer’s voltage reading. The Arduino can control the time interval and frequency of the test patterns’ generation. The voltage of the test pattern can be adjusted by the potentiometer on the PCB. Moreover, the MOSFET on the PCB uses an active socket, allowing for the replacement of MOSFET. Multiple adjustable parameters can be optimized according to the type of strip, ensuring that the measurement parameters fall within the optimal range.

Several characteristic tests were performed on the PCB, including varying the load resistance and gate voltage, and also adding an external capacitor parallel to the MOSFET. The heightened concentration of the sample solution induced an elevation in capacitance, elucidated by the electric double layer theory [32]. Consequently, commercial external capacitors were used as the strip during the test to determine the optimal operational settings.

## 3. Results and Discussion

**Figure 5:**
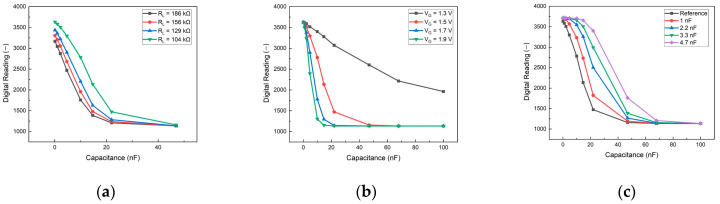
Effect of capacitance on the board. (**a**) Various levels of load resistance with a fixed gate voltage (1.5 V); (**b**) various gate voltages with a fixed load resistance (104 kΩ); (**c**) addition of an external capacitor parallel to the MOSFET, setting R_L_ = 104 kΩ and V_G_ = 1.5 V as a reference.

Tests were performed on the PCB, adjusting the load resistance and gate voltage, and adding an external capacitor parallel to the MOSFET. The outcomes of these tests are illustrated in Figure 5. These results served as a guide for optimizing the board’s conditions to enhance the sensitivity of our detection method. In Figure 5a, the impact of adjusting the load resistance connected to the supply voltage VDD on the PCB is demonstrated. A lower load resistance created more space for the voltage drop, expanding the operational range for testing and enhancing sensitivity. However, due to constraints imposed by other components on the PCB, the lowest permissible condition was set at 104 kΩ. Consequently, 104 kΩ was selected as the load resistance for subsequent tests. Figure 5b presents the device’s performance under varying gate voltages. The sensitivity increased with higher gate voltages, and although V_G_ = 1.9 V exhibited the steepest slope for optimal sensitivity, it yielded a smaller detection range compared with other gate voltages. Striking a balance between sensitivity and detection range, V_G_ = 1.5 V was used in subsequent biomarker tests. The outcomes of incorporating an external capacitor parallel to the MOSFET are depicted in Figure 5c. The total capacitance ($C_{total}$) of the strips and MOSFET can be expressed as

(1) $$\frac{1}{C_{total}}=\frac{1}{\left(C_{MOSFET}+C_{external}\right)}+\frac{1}{C_{strip}}$$

where $C_{MOSFET}$ is the MOSFET’s capacitance, $C_{external}$ is the external capacitor’s capacitance, and $C_{strip}$ is the strip’s capacitance. Notably, the digital reading exhibited significant differences only when the MOSFET’s capacitance approached that of the strips. Consequently, when the sample’s capacitance was substantial, the external capacitor proved beneficial in extending the detection range towards larger values of capacitance.

**Figure 6:**
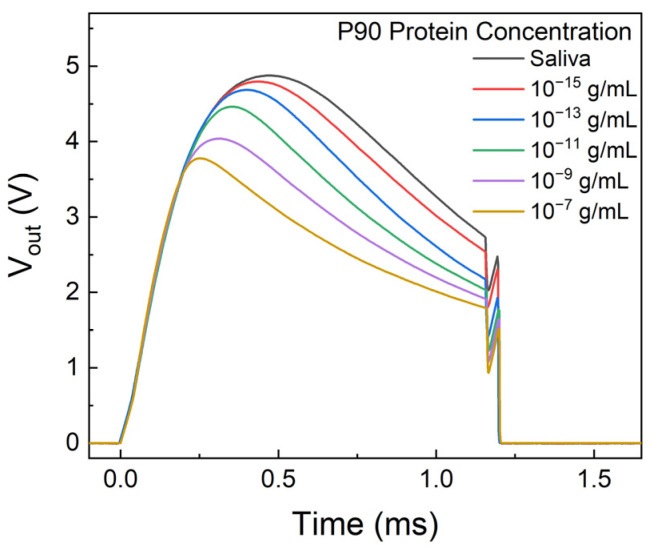
Output voltage pulse from the PCB with different concentrations of P90 protein.

The output voltage from the printed circuit board (PCB) for standard solutions with a series of P90 concentration is depicted in Figure 6. The pattern of the voltage pulse can be elucidated by the double spring model [33].

**Figure 7:**
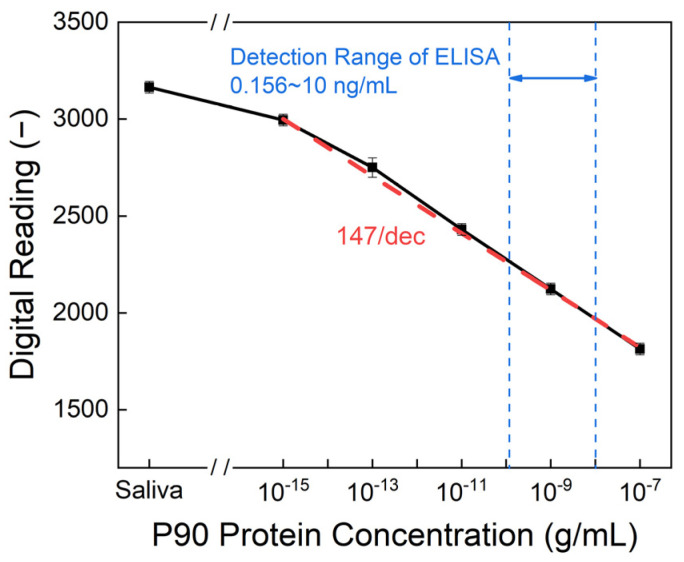
Output of the digital reading from the PCB under different concentrations of the P90 protein. The limit of detection was 10^−15^ g/mL, while the sensitivity was 147/dec.

The digital output reading was obtained by integrating the area under the curve of the output voltage. Figure 7 illustrates the calibration curve, showcasing a sensitivity of 147/dec. This implies that the digital reading decreased by approximately 147 when the protein concentration increased by one order of magnitude. In addition to its commendable sensitivity, the detection method achieved a limit of detection (LOD) as low as 10^−15^ g/mL, while the detection range of commercial ELISA kits is limited to 0.156 to 10 ng/mL [24]. Furthermore, according to the literature, Ding et al. used vertically aligned carbon nanotube array coatings for oral cancer screening using the P90 protein. Their detection range was around 1–100 pg/mL, with an LOD of 0.24 pg/mL. Our sensor performed much better than those reported in the literature [28].

**Figure 8:**
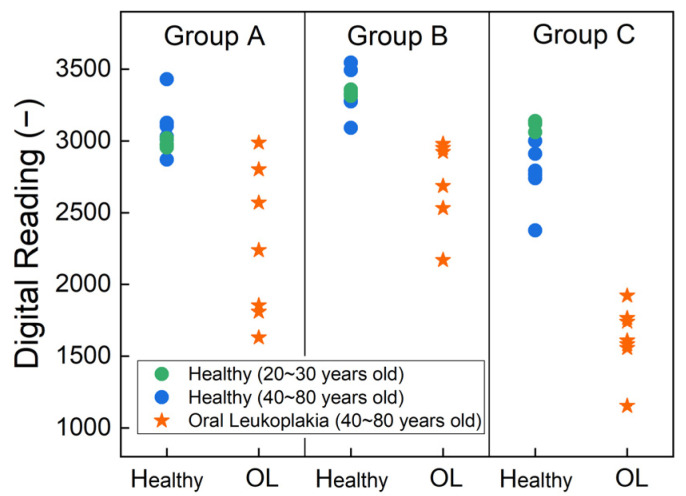
The output of the digital reading of the results from the human sample test with strips functionalized by P90 antibodies. Saliva and tissue samples were tested after cell lysis. Group A represents the result for saliva without cell lysis. Group B was tested with lysed saliva, while Group C was tested with lysed tissue samples.

In addition to evaluating the standard solution, human sample testing was conducted in this study. Figure 8 illustrates the results of the human sample test with saliva and tissue samples after the process of cell lysis. We conducted experiments on three distinct sample groups. Group A comprises saliva samples that did not undergo the process of cell lysis. Group B underwent testing after lysis of the saliva, whereas Group C consisted of tissue samples subjected to lysis before testing. We used different colors to distinguish the age ranges in the figure: green dots represent data from healthy volunteers aged 20 to 30 years, while blue dots represent individuals aged 40 to 80 years. Across Groups A, B, and C, there was no significant difference in the expression of P90, consistent with Bockelman et al.’s findings [34]. Notably, Group A utilized distinct PCB settings compared with Groups B and C, as determined by the earlier capacitance study, aimed at optimizing the sensitivity of detection.

**Figure 9:**
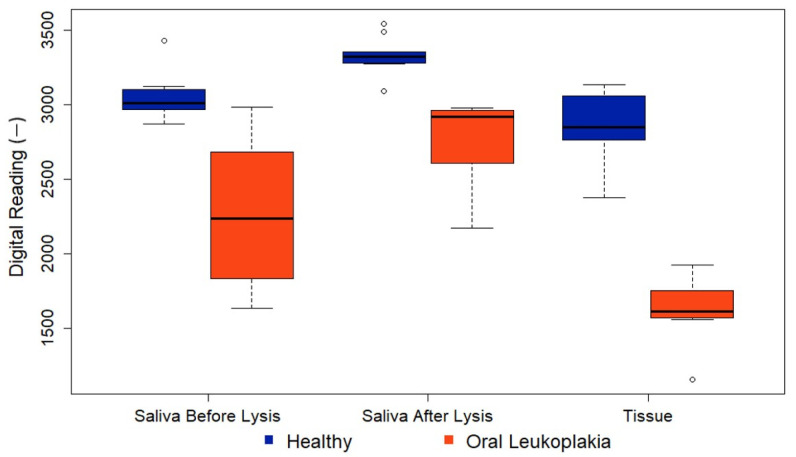
Boxplot depicting the distribution of the test results.

Figure 9 illustrates a boxplot representing the distribution of the test results. Within the boxplot, the bottom corresponds to the 25th percentile (Q1), the middle line indicates the median, and the top of the box signifies the 75th percentile (Q3). The upper and lower branches represent the maximum and minimum values of the data, respectively, excluding outliers. Outliers are depicted as individual data points. An outlier, which is represented as a hollow dot in the figure, is defined as a data point that falls significantly outside the expected variation around the median of the remaining dataset. For the interpretation of the first column, focusing on the healthy group, the readings of the saliva before lysis showed a median of just over 3000, with Q1 at approximately 2950 and Q3 at about 3150. The highest non-outlier observation was around 3200, while the lowest was approximately 2800. Notably, there was one observation at around 3450, which stands out as unusually high compared with the tightly clustered data around 3000. Thus, we defined this as an outlier. Similar interpretations apply to the second and third columns.

In Group A, saliva samples were analyzed without cell lysis, revealing potential differences indicated by the lower readings in pre-cancer samples and a significant *p*-value of 0.001. The *p*-values were derived from Fisher’s exact tests for categorical variables and Mann–Whitney tests for continuous variables. Groups B and C had identical PCB settings to ensure comparable readings. As expected, all data points in Group C were lower than those in Group B, indicating a higher P90 concentration in the tissue samples. The *p*-value for Group B was 0.0001, demonstrating the efficacy of the technique in a non-invasive manner. Furthermore, for Group C, the *p*-value decreased even further to 0.0008. The low *p*-value indicated the low probability of a false positive result, underscoring the technique’s high accuracy. Detailed findings of analyzing the data are summarized in Table 2.

Utilizing the calibration curve derived from the standard solution and the results of the human sample tests, we could integrate these findings to estimate the relative protein concentration in the human samples. Demonstrating a lower LOD compared with commercial ELISA kits, the sensor introduced in this study exhibits the capability to differentiate low-concentration samples and provides valuable insights for oral cancer detection. Furthermore, the biosensor in this study is cost-effective compared with other commercially available screening tests. Examinations for screening oral cancer typically cost around $70–90 USD [35]. In contrast, the disposable sensor strips in this study cost only a few cents each, while the reusable PCB costs around $5 USD. With mass production, the cost could be even lower, making it a very suitable device for future point-of-care applications.

## 4. Conclusions

In conclusion, this study marks a significant advancement in the realm of oral cancer diagnostics by introducing a transistor-based biosensor system for the detection of the P90 protein; this marker is involved in inflammatory processes and with oral squamous cell carcinoma. Our data indicated that P90 may be associated with leukoplakia that may have a potential for progression to malignant oral transformation. The specially designed printed circuit board (PCB) optimizes the functionality of the biosensor, ensuring accurate measurements and enhanced sensitivity. The superior sensitivity, the low limit of detection, and successful human sample testing underscore the potential of this biosensor technique as an invaluable tool in the early detection of oral cancer, ultimately contributing to improved patient outcomes and a reduction in the global burden of this prevalent and impactful disease. The findings presented in this study mark a positive step forward in the ongoing efforts to enhance diagnostic accuracy and efficacy in the management of oral cancer.

## Figures and Tables

**Table 1:** Patients’ characteristics. The *p*-values are the results of Fisher’s exact tests (categorical variables) and Mann–Whitney tests (continuous variables).

		Healthy (*N* = 10, 59%)	Oral Leukoplakia (*N* = 7, 41%)	*p*-Value
Gender	Male	6 (60%)	3 (43%)	0.637
	Female	4 (40%)	4 (57%)	
Age	20–30	3 (30%)	0 (0%)	0.045
	40–60	5 (50%)	2 (29%)	
	60–80	2 (20%)	5 (71%)	
Race	Asian	4 (40%)	1 (14%)	0.338
	White	6 (60%)	6 (86%)	

**Table 2:** Analytical results of the test. Continuous variables presented as the mean (standard deviation), median [interquartile range], or range. Categorical variables presented as N (row%). The *p*-values are the results of Fisher’s exact tests (categorical variables) and Mann–Whitney tests (continuous variables).

	Healthy (*N* = 10, 59%)	Oral Leukoplakia (*N* = 7, 41%)	*p*-Value
Group A	3049 (152); 3014 [2972, 3084]; (2870, 3430)	2270 (529); 2238 (1832, 2685]; (1630,2987)	0.001
Group B	3336 (123); 3326 [3288, 3358]; (3091, 3545)	2745 (306); 2922 [2608, 2964]; (2170,2979)	0.0001
Group C	2867 (230); 2850 [2766, 3046]; (2376, 3138)	1618 (241); 1609 [1570, 1752]; (1154,1921)	0.0008

## Data Availability

The data that support the findings of this study are available within the article.
